# Pseudotime Dynamics in Melanoma Single-Cell Transcriptomes Reveals Different Mechanisms of Tumor Progression

**DOI:** 10.3390/biology7020023

**Published:** 2018-04-03

**Authors:** Henry Loeffler-Wirth, Hans Binder, Edith Willscher, Tobias Gerber, Manfred Kunz

**Affiliations:** 1 Interdisciplinary Center for Bioinformatics, University of Leipzig, 04107 Leipzig, Germany; wirth@izbi.uni-leipzig.de (H.L.W.); binder@izbi.uni-leipzig.de (H.B.); willscher@izbi.uni-leipzig.de (E.W.); 2 Department of Evolutionary Genetics, Max Planck Institute for Evolutionary Anthropology Leipzig, 04103 Leipzig, Germany; tobias_gerber@eva.mpg.de; 3 Department of Dermatology, Venereology and Allergology, University of Leipzig, 04103 Leipzig, Germany

**Keywords:** single-cell transcriptomics, melanoma, pseudotime, tumor progression, gene signatures

## Abstract

Single-cell transcriptomics has been used for analysis of heterogeneous populations of cells during developmental processes and for analysis of tumor cell heterogeneity. More recently, analysis of pseudotime (PT) dynamics of heterogeneous cell populations has been established as a powerful concept to study developmental processes. Here we perform PT analysis of 3 melanoma short-term cultures with different genetic backgrounds to study specific and concordant properties of PT dynamics of selected cellular programs with impact on melanoma progression. Overall, in our setting of melanoma cells PT dynamics towards higher tumor malignancy appears to be largely driven by cell cycle genes. Single cells of all three short-term cultures show a bipolar expression of microphthalmia-associated transcription factor (MITF) and AXL receptor tyrosine kinase (AXL) signatures. Furthermore, opposing gene expression changes are observed for genes regulated by epigenetic mechanisms suggesting epigenetic reprogramming during melanoma progression. The three melanoma short-term cultures show common themes of PT dynamics such as a stromal signature at initiation, bipolar expression of the MITF/AXL signature and opposing regulation of poised and activated promoters. Differences are observed at the late stage of PT dynamics with high, low or intermediate MITF and anticorrelated AXL signatures. These findings may help to identify targets for interference at different stages of tumor progression.

## 1. Introduction

Melanoma is a highly aggressive tumor of the skin and accounts for the majority of deaths from skin cancer. There is an increasing incidence with a current rate of 15/100000 inhabitants per year in Northern European and Northern American countries. Treatment of metastatic melanoma targeting genetically activated oncogene pathways (BRAF/NRAS/KIT pathways) and so-called immune-checkpoints have significantly improved overall survival rates of metastatic melanoma patients in recent years [1,2]. Targeted treatment of activated oncogenes is mainly directed against mutant BRAF (present in 40–50% of all melanomas) using the small molecule inhibitors vemurafenib, dabrafenib and encorafenib. However, recurrence rates due to secondary resistance affect the vast majority of patients. More recent studies have shown that combination treatments of BRAF and its immediate downstream kinases, MEK1/2, are significantly more effective than BRAF-inhibitor treatment alone [3]. However, even among the combined treatment, half of the patients progress after several months [4].

Molecular heterogeneity has been described for a significant number of cancers and is regarded as a major mechanism for poor treatment response, treatment resistance and early recurrence after treatment [5]. Based on a series of recent studies, melanoma has a high inter-tumor and intra-tumor heterogeneity [6,7,8,9]. Thus, analysis of the subclonal structure may help to better understand and improve treatment approaches. Recent progress in single-cell sequencing technology allows for a more detailed understanding of tumor heterogeneity and clonality by use of single-cell transcriptomics [10]. A large series of reports using this technology have provided deeper insight into the clonal structure of different cancers [11]. Two studies, one own study, and one from an independent group, have recently been published using genome-wide single-cell transcriptomics for either melanoma short-term cultures or melanoma tissues [12,13]. Here, we further exploit this data by analysis of pseudotime (PT) dynamics to characterize tumor heterogeneity and to find indications for different (e.g., bipolar, divergent, parallel, switch-like) modes of gene expression during tumor progression, which might reveal new targets for therapeutic interference. 

Our PT-analysis is motivated by the fact that human cancer is an inherently dynamic disease that develops over an extended time period through the accumulation of a series of genetic and/or epigenetic defects disturbing genomic regulation of normal cells. Cancer development can be viewed as Darwinian evolutionary process at the cellular level driven by (epi-)genetic variations leading to a heterogeneous distribution of cellular phenotypes and a selective process shaped by the microenvironment, treatment and other factors [14]. The study of cancer developmental dynamics requires time-course studies by repeated sampling of the same cohort of subjects. However, due to the need for immediate treatment upon diagnosis, and other reasons this approach is not feasible in most situations and one has to rely on cross-sectional data collected from different patients and by assuming that each tumor is an independent realization of the same evolutionary process. Such static sample data provide a snapshot of the disease process where the individual samples populate the developmental progression trajectory. Tissue sampling however provides only a mean picture averaged over all cellular states present in the tumor sample, which potentially masks cell-specific molecular mechanisms. Modern single cell sampling and sequencing techniques promised to overcome this problem. However, also single-cell RNA-seq produces only a static view of gene expression within cells. Computational PT-methods open an option for studying temporal processes in cross-sectional gene expression data by making the assumption that cells at various stages of development are present in one RNA-seq dataset and map onto PT by applying criteria of mutual similarity between their transcriptional states. Hereby, the ‘pseudotime’ is a quantitative, often one-dimensional measure of progress through a biological process along the cells are arranged. Applying PT-methods to cancer data allow to track genes and associated gene-regulatory programs activated at various stages of cancer progression on cellular level, e.g., from normal tissues to malignant lesions.

Our recent single cell study resolved the heterogeneity of cellular states in cultures of melanoma cells which harbor either BRAF or NRAS mutations, or none of them. Particularly we identified a series of transcriptional modules of co-regulated genes involved in proliferation, oxidative phosphorylation, pigmentation, cellular stroma, ABC-transporter activity and others [12]. By comparison of our data with in vivo melanoma single cell expression signatures, we could show that in vivo properties were conserved in in our vitro cultures. We could also show that the expression programs activated in different groups of melanoma cells correspond to expression signatures of melanoma tumors from different progression stages including healthy skin, low grade, high grade and metastatic tumors. Our conclusion that the respective cellular programs reflect different developmental stages with increasing aggressiveness was further supported by comparison of our single cell data with expression signatures of embryonic stem cell (ESC) and neural progenitor cells (NPC), characterizing poorly differentiated tumors and also with signatures of a series of chromatin states in fibroblasts and melanocytes characterizing benign states. We found that the expression signatures of poised and repressed genes in ESCs, melanocytes (MCs), NPCs and partly also in fibroblasts are selectively activated in the melanoma cells studied which were assigned to early tumor stages. This result indicates that these genes act as potential regulators for fate decisions ensuring the developmental plasticity and pluripotency of developing tumor cells in agreement with concepts of epigenetic mechanisms of tumor development [15,16]. We also found indications for deregulation of these states due to epigenetic mechanisms leading to cellular reprogramming in later stages of melanoma progression. Taken together, our previous results imply and justify the application of PT methods to study potential mechanism of melanoma development in more detail and particularly to disentangle common and different properties of tumor progression dynamics associated with BRAF- and NRAS-mutations.

## 2. Materials and Methods

### 2.1. Ethics Approval and Consent to Participate

The study has been approved by the Ethics committee of the Medical Faculty of the University of Leipzig (Az 023-16-01022016). The study was conducted according to the Declaration of Helsinki.

### 2.2. Short-Term Cultures

Single-cell RNA-Seq data were derived from an own study on three melanoma short-term cultures (BRAF wild type (wt)/NRAS-wt, BRAF mutant (mut)/NRAS-wt and BRAF-wt/NRAS-mut, respectively) [12]. BRAF and NRAS mutations are mutually exclusive in melanoma. Except very rare cases, a patient is either BRAF or NRAS-mut or wild type for both [17]. The mutational status was re-confirmed immediately before the current experiments. The mutant short-term cultures of our study reflect the most prominent mutations in melanoma (BRAF mutations in 52%, NRAS mutations in 28%, BRAF-wt/NRAS-wt in 20%) [8]. The BRAF-mut status reflects the most prevalent somatic mutation of the V600E amino acid residue while the NRAS-mut status refers to the less common mutation G13R [8]. The single cells thus consider frequent mutations of melanoma cells. The impact of this cell system for melanoma tumors and, particularly, the correspondence between activated cellular programs and melanoma progression stages has been judged by applying melanoma specific gene signatures to the data in our previous publication [12]. Melanoma cells were cultured under standard conditions in two-dimensional (2D-) dishes as described [17]. Melanoma cells were kept for several passages in cultures before use in the RNA-seq experiments, which removed contaminating cells such as immune cells. A contamination with fibroblasts was ruled out by testing for expression of fibroblast specific genes.

### 2.3. Single-Cell RNA-seq and Data Preprocessing

Sequencing protocol was previously described in [12]. In brief: Single melanoma cells from short-term cultures were captured on an integrated fluidic circuit RNA-seq chip (Fluidigm, Hamburg, Germany) using the Fluidigm C1 system. Cell capture, cell lysis, reverse transcription, and cDNA amplification were performed according to standard procedure [18]. Illumina libraries were constructed using the Illumina Nextera XT DNA Sample Preparation kit using the protocol supplied by Fluidigm. Library concentration and size distribution were assessed on an Agilent Bioanalyzer and with Qubit dsDNA HS Assay kits and a Qubit 2.0 Fluorometer (Thermo Fisher Scientific, Invitrogen, Darmstadt). Each cell was paired-end sequenced (100 base reads) on an Illumina HiSeq 2500 (Fluidigm).

Raw reads were processed using standard tools and default settings: Bowtie2 [19], TopHat [20], and Cufflinks [21]. Transcript levels were quantified as fragments per kilobase of mapped reads (FPKM). Utilization of FPKM values normalizes read counts for both sequencing depth and length of the gene. Data was then corrected for cell cycle status of the cells using Cyclone tool as described in [22]. This correction virtually removes variance due to cell cycle phases using appropriate cell cycle gene signatures. Finally, effects of total read number across the cells are avoided using quantile normalization, resulting in identical data distributions for all cells. We excluded cells that did express neither of two housekeeping genes ACTB and GAPDH. Eighty-four single cells remained for the transcriptome analysis of the BRAF/NRAS-wt/wt culture, 54 cells for the BRAF-mut/NRAS-wt, and 77 cells for BRAF-wt/NRAS-mut culture, respectively. 

### 2.4. Data Availability

The data is publicly available from Gene Expression Omnibus (GEO) repository under accession number GSE81383.

### 2.5. Pseudotime Analysis

Single-cell gene expression profiling can be used to quantify transcriptional dynamics in temporal processes, such as cell differentiation or cancer progression, using computational methods to label each cell with a pseudotime (PT) where experiments of true time series are unavailable [23]. The PT model assumes that single cell transcriptomes can be understood as a series of microscopic states of cellular development, e.g., of progressing cancer, that exist in parallel at the same (real) time point in the cell culture under study. Furthermore the model assumes that dimension reduction techniques maintain the temporal ordering. Practically this assumption means that state changes should proceed smoothly and continuously in small increments.

One particularly robust method to estimate PT in single-cell data is the Wanderlust algorithm which is used for PT calculation in this study [24]. In brief, this approach generates an ensemble of K-nearest neighbor graphs based on expression similarity relations which include complete gene expression data into downstream analysis. We chose k = 16, which leads to a sufficient number of edges in the graph and a clear structure. Variation of k between 10 and 20 led to very similar graphs. Trajectories are obtained for each graph using all shortest path distances and then calculating its mean which defines the PT [24]. Wanderlust is designed to calculate linear, i.e., non-branched, PT trajectories. We therefore applied it to the data of each short-term culture separately in order to avoid mixing of different genetic backgrounds. Cells showing a high stromal expression signature were assigned to low PT-values according to the similarity with the expression signature of early melanoma stages [12]. Wanderlust then provides a PT score for each single cell in intervals (0, 1). Cells with high proliferative activity were assigned to high PT values according to similarities with the expression signature of late stages of melanoma progression (vide supra). Stromal and proliferative signatures of the single cell data were shown to change antagonistically upon development of melanoma from healthy skin via low grade, high grade towards metastatic melanomas [12] which justifies the PT as a proxy for progressing tumor. PT thus is assumed to sort the cells according to the intended developmental stage, but it does not provide a temporal unit that quantifies the time span of the processes under study.

### 2.6. Functional PT-Profiles

For functional analysis we used a series of pre-defined gene sets with known functional contexts taken from data repositories and previous publications [13,25,26,27,28,29]. The activity of a gene set in each cell was estimated using the gene set Z-score (GSZ) introduced by Törönen and co-workers [29] and used by us as described previously [30]. Functional PT-profiles were then obtained by smoothing the PT-ranked GSZ values of the cells using LOESS (local weighted scatterplot smoothing). For direct comparison between the functional profiles, we calculated a relative activity of the respective cellular program by normalizing the smoothed GSZ-profiles between the maximum and minimum values of each profile which were set to unity and zero, respectively. LOESS-smoothing was also applied to bi-variate plots to obtain the trajectories of pairwise combinations of gene sets.

## 3. Results

### 3.1. RNA-seq Based Pseudotime Dynamics in Patient-Derived Melanoma Cell Cultures

**Figure 1:**
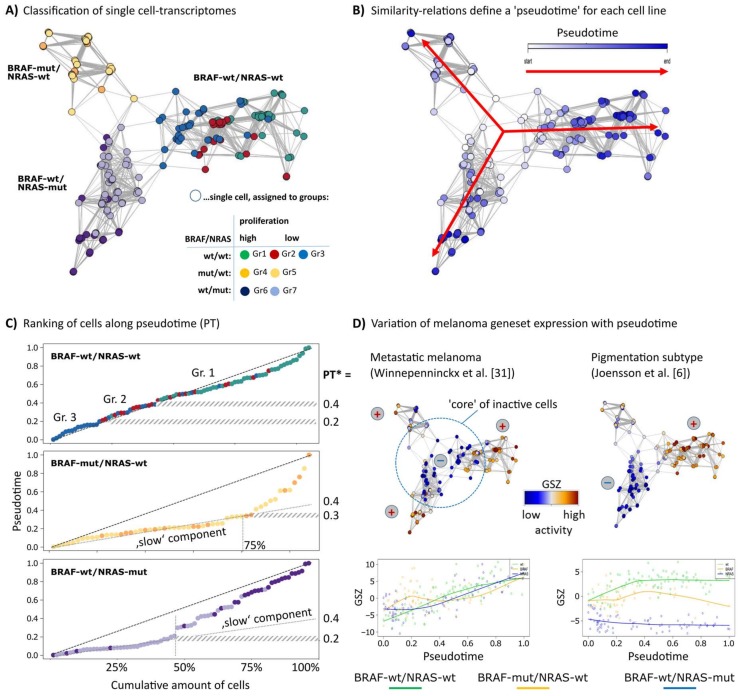
Diversity and pseudotime (PT) ranking of single-cell transcriptomes of melanoma cell short-term cultures. An analysis of single-cell transcriptomes of melanoma short-term cultures was performed using the Wanderlust algorithm. (**A**) The network presentation visualizes similarity relations between the single-cell transcriptomes of the three cell cultures studied. Each cell culture forms a separate branch where the cells were classified into seven groups as described previously [12]. (**B**) Cells were recolored according to their PT dynamics from early (white) to late (dark blue) in direction of the arrows where the highly proliferative cells were assigned to later PT. The PT was calculated separately for each cell line. (**C**) The plots of PT as a function of call rank indicate a virtually linear relation for wt/wt cells or an initial slow component for the two mutant cell lines. PT* defines characteristic PT-values: PT’s referring to alterations of the group-programs (BRAF-wt/NRAS-wt) and alteration from the the ‘slow’ into the ‘faster’ component (BRAF-wt/NRAS-mut and BRAF-mut/NRAS-wt). (**D**) Mean expression (GSZ-score) of signature gene sets of metastatic melanomas described by Winnepenninckx and co-workers [31] is consistently high at late PT values in all cell lines, which indicates that PT direction is consistent with melanoma progression towards high grade (bad prognosis) melanomas. Signature genes of the pigmentation subtype [6] show a bipolar pattern with high expression in wt/wt cells, low expression in BRAF-wt/NRAS-mut cells and intermediate and fluctuating levels in BRAF-mut/wt cells.

Single-cell RNA-seq data of three melanoma short-term cultures of BRAF/NRAS double wild type (wt/wt), BRAF-mutant/NRAS-wild type (BRAF-mut/NRAS-wt) and BRAF wild type/NRAS-mutant (BRAF-wt/NRAS-mut) cells were analyzed regarding PT dynamics to assess mechanisms of tumor progression. Each cell culture arranges as a separate branch in the network presentation (Figure 1A). The cells were classified into seven groups as described previously [12]. Groups 1, 4 and 6 comprise highly proliferative cells. Groups 2 and 3 of wt/wt cells showed pigmentation and stromal gene expression signatures, respectively, and groups 5 and 7 showed signatures specific for BRAF- or NRAS-mutant cells [12]. The cells of each cell culture were sorted in one dimension based on mutual similarities of their transcriptomes using the Wanderlust algorithm (Figure 1B) [24]. This algorithm assumes that single-cell transcriptomes of cell cultures or tissues in principle constitute a time series in which each cell represents a distinct time point along a continuum, representing an underlying mode of temporal progress. Thus, assignment of a PT to each cell provides a relative quantitative similarity measure of developmental progression. The plots of the PT as a function of the elapsed PT reveal an almost linear relation for the wt/wt cells where the cells arrange in a way according to their group assignments of group 3/stromal (first) and group 1/proliferating (last) (Figure 1C). The BRAF-mut/NRAS-wt and BRAF-wt/NRAS-mut cell cultures show a bimodal course of the PT with a flat, initial slope indicating that about 50%/75% of the cells are relatively similar to each other. This slow component refers to a typical PT threshold of PT* = 0.2 and 0.3, respectively, and suggests a switch in cell activity which will be described below.

To relate the single-cell PT data to published melanoma gene expression signatures, we calculated the mean expression of sets of melanoma gene signatures for each cell as a function of PT (Figure 1D). Gene signatures of high grade (bad prognosis) melanomas taken from an earlier study on primary melanoma samples steadily increased their expression in the cells along the course of PT, with highest expression at late PT values in all three cell cultures [31]. This result suggests that PT dynamics correlates with tumor progression towards higher tumor grades, independent of mutational status. Gene expression signatures of primary melanomas with poor prognosis from an independent study showed similar results (data not shown) [7]. A signature characterizing the pigmentation subtype of melanomas shows a bipolar expression pattern with high activity in the wt/wt cell culture and low activity in the BRAF-wt/NRAS-mut cell culture, and a fluctuating expression in BRAF-mut/NRAS-wt cells [6] (Figure 1D). These results indicate that the PT dynamics of the three different cell cultures reflect gene expression signatures from melanoma tissues in vivo with individual characteristics for each cell culture.

### 3.2. Cell-Culture Specific Dynamics of Functional Signatures 

To further interpret PT in terms of functional categories of the underlying cellular programs we extracted gene sets varying in concert with PT or contrary to PT using likelihood ratio test of a generalized additive model (GAM), with PT as co-factor against a reduced model with no PT dependence [32]. Gene sets related to cell division and proliferation and mitochondrion-related processes correlated with PT progression in wt/wt and BRAF-mut/NRAS-wt cells, and BRAF-wt/NRAS-mut cells, respectively (Table 1). 

**Figure 2:**
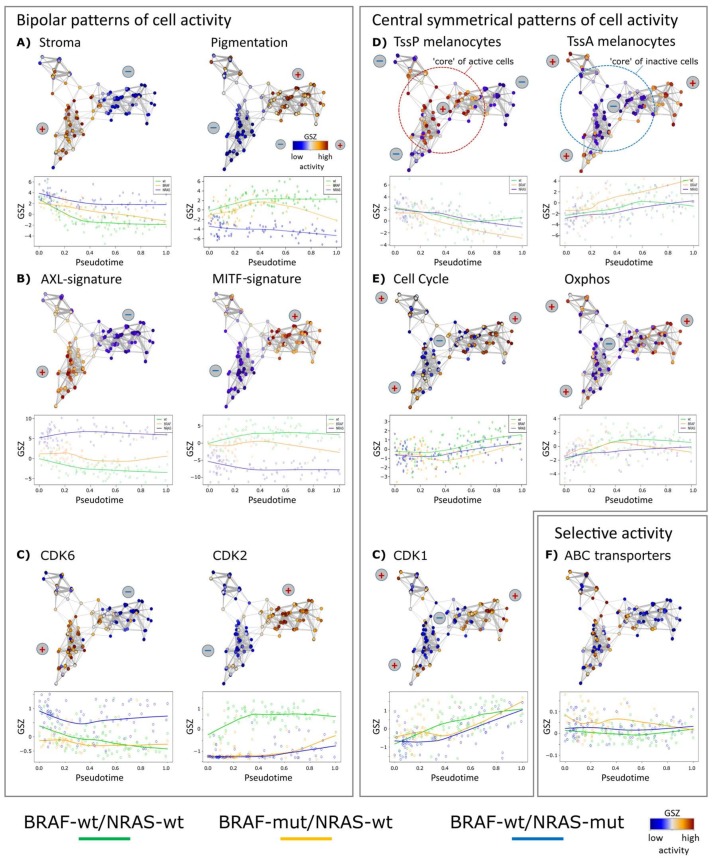
Changes of melanoma expression signatures with PT. Melanoma short-term cultures were analyzed by single-cell RNA-seq and subjected to PT analysis using Wanderlust algorithm. (**A**) Stromal and pigmentation signature. (**B**) AXL and MITF signatures show a virtually bipolar characteristics with high levels of AXL in the NRAS-mut cells and low levels in the BRAF-mut and wt/wt cells, respectively, also indicated by the plus and minus signs. MITF shows opposite characteristics. (**C**) Gene expression levels of cyclin-dependent kinases CDK6 and CDK2 show bipolar, CDK1 central symmetrical activation patterns, respectively. (**D**) Mean expression of genes attributed to poised (TssP) promoters decreased with PT, while that with active (TssA) promoters increased. (**E**) Expression of genes related to cell cycle and oxidative phosphorylation (oxphos) both increased with PT, however, in a bimodal way with a delay of cell cycle activity and a plateau phase of oxphos. Gene sets were taken from gene ontology [26] and hallmarks of cancer [25] repositories and from two further studies on stromal and MITF/AXL signatures, respectively [13,27]. (**F**) ABC-transporter genes are selectively expressed across the melanoma cells in agreement with [12].

We analyzed PT dynamics of further functional gene signatures [6,12,13] (Figure 2). Common and disparate features were identified in the different cell cultures. All three cultures had a core (early PT) program of stromal (extracellular matrix) genes, which decreased during PT progression (Figure 2A). Among the genes of the stromal signature were ANXA1/2 (annexin1/2), FN1 (fibronectin 1), CALD1 (caldesmon 1) and SORBS2 (sorbin and SH3 Domain Containing 2), well-known for their involvement in extracellular matrix interaction of cancer cells. 

Signature genes characterizing a pigmentation (MITF high) subtype of melanomas taken from the study of Jönsson and coworkers show bipolar activation patterns in the present study with high levels in wt/wt cells and low levels in BRAF-wt/NRAS-mut cells and an intermediate, fluctuating levels in BRAF-mut/NRAS-wt cells [6] (Figure 2B). Thus, high proliferation signatures of high grade melanomas (late PT cells) may be associated with high, intermediate or low pigmentation signatures and MITF signatures, suggesting overlapping but also individual patterns of tumor progression. A MITF-low proliferative group has also been described in a recent survey of different melanoma gene expression studies [33]. A bipolar expression characteristic for the three cell cultures was observed for AXL/MITF signature genes in agreement with recent melanoma single cell transcriptome analyses [13]. The MITF signature included cadherin 1 (CDH1), premelanosome protein (PMEL), tyrosinase (TYR), and melan-A (MLANA) and S100 calcium-binding protein B (S100B). The AXL signature included FOS-like 1 (FOSL1), ras homolog family member C (RHOC), nerve growth factor receptor (NGFR), and superoxide dismutase 2 (SOD2).

A common mechanism among the three tested cell cultures was the regulation of chromatin states [34]. The mean activity of poised chromatin states in melanocytes (TssP/poised transcriptional start sites) decreased over PT, while the activity of active promoters (TssA/active transcriptional start sites) increased [35] (Figure 2D). These trends suggest that epigenetic reprogramming of gene activity accompanies or even drives melanoma progression. In line with this, we have recently described marked differences of the activity of chromatin modifying enzymes in subpopulations of the mentioned cell cultures [12], which supports the hypothesis that epigenetic modifications affect melanoma progression. 

Interestingly, PT dynamics of the cell cycle signature shows an initial delay but then a constant increase (Figure 2E). In contrast, the oxphos signature rapidly reaches a plateau indicative of oxphos activation during early stages of cellular proliferation (indicated as cell cycle). In BRAF-mutant cells, a fluctuating expression pattern was found for oxphos, which will be described in more detail below. Selected cell cycle dependent kinases (CDKs) show either bipolar (CDK6 and CDK2) or central symmetrical (CDK1) activation patterns (Figure 2C) where the bipolar patterns show high expression for CDK6 in BRAF-wt/NRAS-mut or CDK2 in BRAF-wt/NRAS-wt cells, respectively. Thus, CDK6 expression parallels that of the stromal and AXL-signatures, and CDK2 expression parallels that of the pigmentation and MITF-signatures, respectively. CDK1 associates with the proliferative signature [12]. ABC transporters were upregulated in about 10% of the BRAF-wt/NRAS-wt cells with possible impact for therapy resistance [12], however without clear trends in PT dynamics (Figure 2F). 

Taken together, PT dynamics of individual gene pattern provided evidence for common and disparate mechanisms of PT progression in different melanoma samples.

### 3.3. High Resolution PT-Dependent Activity of Cellular Programs

**Figure 3:**
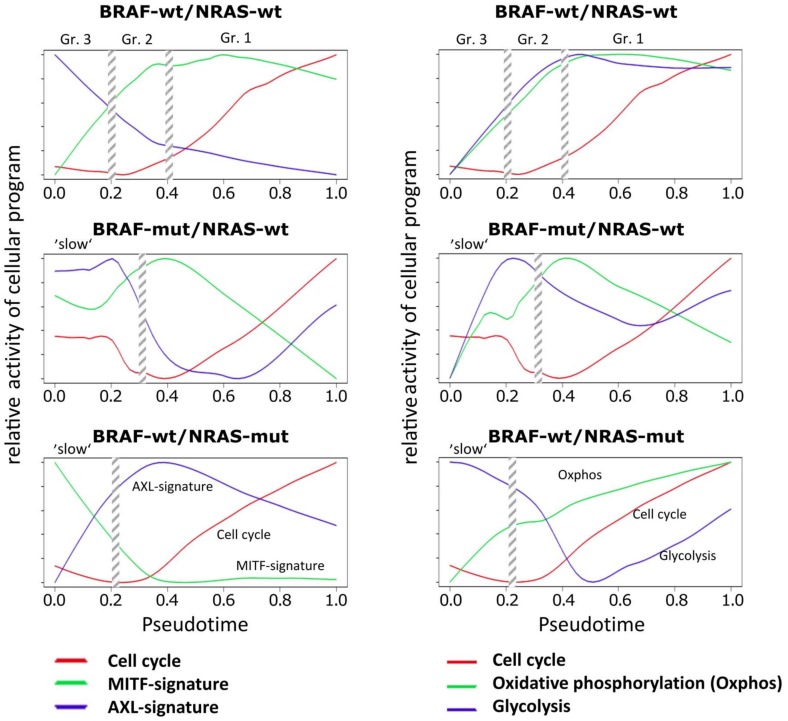
Relative activity of cellular programs of cell cycle, MITF, AXL, oxphos and glycolysis signatures along PT. Melanoma short-term cultures were analyzed by single-cell RNA-seq and subjected to PT analysis using the Wanderlust algorithm and analysis of relative reactivity profiles of cell cycle, MITF, AXL, oxphos and glycolysis signatures. Vertical streaked bars indicate the boundaries of the three different cell subpopulations in wt/wt cells or the two different cell populations in BRAF-mut/NRAS-wt and BRAF-wt/NRAS-mut cells as indicated in Figure 1C.

Next, we transformed the PT-profiles of selected functional categories into relative activity profiles to better resolve details of sequential activation and de-activation of cellular programs with PT in the three cell cultures (Figure 3). A common feature of all cell cultures is a monotonous and slow gain of proliferative (cell cycle) activity after an initial delay which corresponds to the slow phase of PT in the BRAF-mut/NRAS-wt and BRAF-wt/NRAS-mut cells and to the cells of groups 3 and 2 in the wt/wt cell culture.

A second feature observed in all three cell cultures is the gain of oxphos activity which preceded the increase of proliferation (cell cycle). However, oxphos patterns showed a decrease in BRAF-mut/NRAS-wt cells at late PT (Figure 3). Increasing proliferation in these cells may at later stages be supported by aerobic glycolysis in addition to oxidative phosphorylation (oxphos genes). In support of this, aerobic glycolysis gene signatures were upregulated at late PT of BRAF-mut/NRAS-wt cells (Figure 3). Similar findings were observed in BRAF-wt/NRAS-mut cells with slowly increasing oxphos genes and delayed activation of glycolysis genes, while parallel activation was observed in wt/wt cells (Figure 3), suggesting that aerobic glycolysis is also active in these cell cultures. Expression of transcription factor CREB1, a transcription factor known to be involved in the expression of glycolytic enzymes, either preceded the high glycolysis signature in wt/wt cells or preceded its re-induction in BRAF-mut/NRAS-wt and BRAF-wt/NRAS-mut cells (Supplementary Figure S1). 

This analysis of PT dynamics (Figure 3) also showed that the MITF signature clearly preceded the cell cycle signatures in wt/wt and BRAF-mut/NRAS-wt cells, while the AXL signature preceded the cell cycle signature in BRAF-wt/NRAS-mut cells. The downregulation of the MITF signature in BRAF-mut/NRAS-wt cells was associated to upregulation of AXL signature (at late PT). Moreover, the MITF signature paralleled the oxphos signature in wt/wt cells and BRAF-mut/NRAS-wt cells, which is in line with the fact that MITF induces oxphos genes [36]. However, this regulation appears to be decoupled in BRAF-wt/NRAS-mut cells. In these cells downregulation of MITF was accompanied by a slow increase in oxphos signature leading to mid-PT onset of aerobic glycolysis. 

Taken together, the three cell cultures showed common and individual mechanisms of PT progression, and BRAF-mut/NRAS-wt cells appear to use higher cellular plasticity during PT progression regarding MITF/AXL and oxphos/glycolysis signatures. 

### 3.4. Two-Dimensional Trajectories Reveal Different Modes of Mutual Activities of Cellular Programs 

**Figure 4:**
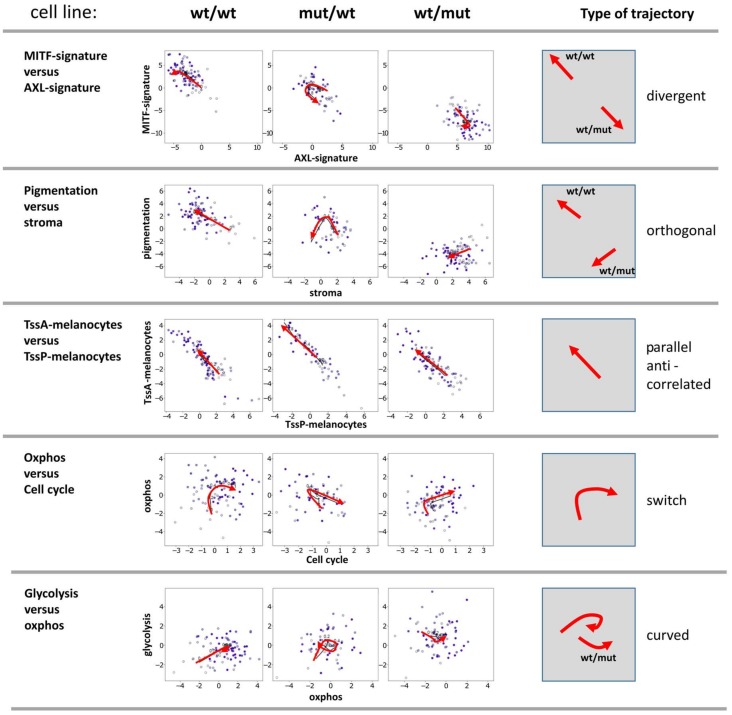
Biaxial trajectories of melanoma progression. Melanoma short-term cultures were analyzed by single-cell RNA-seq and subjected to PT analysis using the Wanderlust algorithm and plotted for biaxial progression trajectories. Plots of pairwise combinations of gene signatures reflect different types of trajectories as schematically indicated in the right part of the figure.

Next, two-dimensional plots of the expression levels of pairwise combinations of functional signatures were generated. These demonstrate biaxial progression trajectories of cellular programs (Figure 4). They illustrate mutual relations between the cellular programs described above. Five prominent pairwise combinations were identified: i) The divergent trajectory of AXL vs. MITF signatures, with an either MITF-low/AXL-high pattern or vice versa, shows the antagonistic nature of these programs which are anti-correlated and change into different directions with PT. BRAF-mut/NRAS-wt cells were able to switch between both states; ii) pigmentation and stromal signatures followed orthogonal trajectories meaning that the activity of the pigmentation signature either increases of decreases with decreasing stromal activity in the wt/wt and BRAF-wt/NRAS-mut cell cultures, respectively; iii) a parallel and anti-correlated trajectory for promoter activity of poised and active promoters (TssP/TssA) reflects the fact that the increase of TssA genes is paralleled by a decrease of TssP genes; iv) a switch-like behavior, where oxphos signatures reaching a plateau switched into proliferation (cell cycle) signatures supporting the above observation that the gain of oxphos activity precedes cellular proliferation in PT; iiv) a curved behavior was observed for genes involved in glycolysis vs oxphos, which means that gene expression signatures are partly correlated but also switch-like (BRAF-mut/NRAS-wt cells). 

The two-dimensional trajectories showed that PT dynamics of the BRAF-mut/NRAS-wt cells more closely resembled that of the wt/wt cells, especially at early PT values. The differences between BRAF-wt/NRAS-mut and the two other cell lines were due to the divergent- and orthogonal-type trajectories at later PT (highly proliferating cells). These different trajectories reflect certain combinatorial patterns of cell activities in the different cell lines where the BRAF-mut/NRAS-wt cells seem to combine properties of wt/wt and NRAS-mut cells.

## 4. Discussion

An analysis of PT dynamics was performed for melanoma short-term cultures to identify genes and gene signatures with different modes of expression during melanoma progression. We identified a progressive loss of extracellular matrix genes during PT towards higher malignancy, a constant bipolar expression for MITF/AXL signatures, an increase in genes expressed from active promoters, and a switch-like expression in oxphos/cell cycle signatures towards late PT. These patterns appear to be relatively common mechanism involved in melanoma progression but with slight modifications, e.g., for MITF/AXL expression and oxphos. Furthermore, different modes of relations between these signatures were found as demonstrated by different trajectories. 

Stromal genes showed a progressive loss of expression during PT progression. Although stromal (matrix) genes play an important role in melanoma biology, no explicit stromal signatures have been described in different earlier gene expression studies on melanoma subtypes with prognostic capacity [6,7,8], which might suggest that stromal signatures are a common/overlapping feature of all melanoma tissues. Interestingly, stromal genes may discriminate between melanoma cell lines and melanoma tissues, although only a small percentage (4.5%) of genes of top differentially expressed genes between both belonged to a stromal signature [37]. In a recent melanoma single-cell study, it was shown that stromal signatures derived from melanoma-associated fibroblasts were also found in whole-tissue gene expression analyses and correlated with the immune-cell infiltration [13]. Whether these stromal gene patterns of whole tissues are derived from fibroblasts alone or also from tumor cells is not completely clear. The impact of melanoma cells themselves on immune surveillance is well-known. Moreover, the loss of expression of stromal genes has been reported for initial tumor development in melanoma [38].

A bipolar expression characteristic for the three cell cultures was also observed for AXL/MITF signature genes. Opposing AXL/MITF expression in melanoma subsets has gained recent interest, because MITF-low/AXL-high expression signatures were found in tumors of a subgroup of melanoma patients resistant to BRAF/MEK inhibitor treatment [39,40]. AXL inhibition by small molecule inhibitors or siRNA was shown to overcome this treatment resistance [40]. A MITF-low subpopulation has been defined in a large-scale RNA-seq analysis of more the 330 melanomas [8]. In this latter study, MITF-low patients had a worse prognosis than patients with so-called immune subclass tumors, but a better prognosis than patients with so-called keratin subclass tumors.

The underlying mechanisms for the opposite regulation of MITF and AXL are as yet poorly understood. Recently, evidence was provided that translational reprogramming under microenvironmental stress, involving the ATF4 transcription factor, may play a role in downregulation of MITF and upregulation of AXL [41]. However, this may not necessarily explain our findings in melanoma cell cultures. Evidence for opposite gene expression characteristics has been described in other studies, but indeed bipolar expression on a single-cell level in different cell cultures has not been definitely shown until our own, and an independent recently published single-cell study provided evidence for this phenomenon [12,13,37]. The transient maximum and later low MITF signature (fluctuating expression) in BRAF-mut/NRAS-wt cells may be due to the fact that highly proliferating cells may well have low MITF expression, defined as a MITF-low proliferative group in a recent meta-analysis of different melanoma gene expression studies [33]. Interestingly, in our setting, MITF downregulation at late PT was accompanied by increasing AXL expression, suggestive for a role of AXL in melanoma cells proliferation in the absence of MITF. The underlying mechanism for this expression pattern suggests a deeper program of MITF/AXL regulation which remains to be analyzed in more detail in future studies.

The mean activity of poised chromatin states (TssP/poised transcriptional start sites) derived from melanocytes decreased over PT, while the activity of active promoters (TssA/active transcriptional start sites) increased [35]. In particular, a reduction of genes with poised promoters (TssP), which include developmental regulators, potentially reduces the options of the cells for fate decisions (at late PT). In contrast, genes with active promoters (TssA) gain activity, which may reflect the need of the cells to support proceeding proliferation by a battery of cellular processes with active genes. 

The increase in oxphos signatures generally preceded the increase of proliferation. In the analysis of two-dimensional trajectories a switch-like behavior was observed where oxphos signatures switched into proliferation (cell cycle) signatures at late PT. This switch was most prominent in BRAF-mut/NRAS-wt cells showing downregulation of oxphos at late PT. Oxphos and aerobic glycolysis are linked via feedback mechanisms [42]. Thus, downregulation of oxphos at late PT was highly suggestive for an activation of aerobic gylcolysis pathway for compensatory energy supply of highly proliferating cells. Indeed, aerobic glycolysis genes were induced in this setting. Evidence has been provided that mutant BRAF may block oxidative phosphorylation [43,44], which in turn may re-activate aerobic glycolysis in specific cell cycle stages. Thus, this may be a particular phenomenon of BRAF-mutant cells when the oncogenic signaling shows high activity. Preceding the glycolysis signature we identified an induction of transcription factor CREB1, which may at least partly contribute to this phenomenon. Indeed, CREB1 is a well-known regulator of glycolytic enzymes hexokinase and lactate dehydrogenase and has recently been shown to be involved in aerobic glycolysis induction in cancer-associated fibroblasts [45].

The MITF signature parallels the oxphos signature in wt/wt and BRAF-mut/NRAS-wt cells, which is in line with the fact that MITF induces oxphos genes [36]. Low MITF expression in BRAF-mut/NRAS-wt cells resulted in slow increase in oxphos signatures, which appears to be compensated by aerobic glycolysis, likely due to a feedback mechanism. 

## 5. Conclusions

Taken together, PT dynamics of individual gene patterns derived from single cell transcriptomics provides an option to study possible mechanisms of melanoma development. It suggests common and disparate mechanisms of PT progression for different melanoma mutants. It further unveiled that MITF/AXL expression inversely correlated on single-cell level which appears to be due to opposing regulatory mechanisms. Moreover, oxphos and cell proliferation signatures are coupled via a switch-like mechanism. Oxphos and glycolysis signatures suggest an extensive growth-promoting and metabolic plasticity in melanoma cells, which should be addressed in further functional studies. PT signatures may point to new mechanisms of therapeutic interference. Application of the PT approach to melanoma tumor transcriptomics data (tissue and single cells) in future studies are required to validate the findings presented here.

## Figures and Tables

**Table 1:** Gene sets and top genes varying in concert with PT.

Cell Line	Top Gene Sets Changing with PT		Top-10 Genes	
BRAF/NRAS	Correlated	Anti-Correlated	Correlated	Anti-Correlated
wt/wt	Sister chromatid cohesion (10^−11^) ^1^	Cell adhesion (10^−10^)	H2AFZ CHCHD6 CD63 TRPM1 MLANA GSTP1 TUBA1B SLC25A5 HSPD1 PMEL (<10^−8^)	ANXA1 CALD1 (<10^−5^) LIMA1 CRYAB FN1 ANXA2 ABL2 FKBP9 A2M ARID5B (<0.002)
	Chromosome, centromeric region (10^−11^)	Ras guanyl-nucleotide exchange factor activity (10^−10^)		
	DNA replication (10^−11^)	Extracellular matrix organization (10^−9^)		
	Kinetochore (10^−11^)	Proteinaceous extracellular matrix (10^−9^)		
	Cell division (10^−10^)	Skeletal muscle tissue development (10^−9^)		
mut/wt	Mitochondrial inner membrane (0.008)	Extracellular matrix organization (0.008)	UQCRHL UQCRH MRPL24 (<0.07) FIBP CCDC167 RAB18 CYC1 KPNA2 RBMX RAB32 (<0.14)	EIF4G3 TUBG1 MITD1 TRIB2 (<0.2) SESN1 TSN EXO1 BIRC5 ITGAE SKA2 (<0.3)
	Mitochondrial respiratory chain complex I assembly (0.01)	Collagen trimer (0.01)		
	Mitochondrion (0.01)	Negative regulation of cell adhesion (0.01)		
	Chromatin (0.01)	Ras guanyl-nucleotide exchange factor activity (0.02)		
	DNA metabolic process (0.01)	Cell adhesion (0.02)		
	Cell division (0.01)			
wt/mut	DNA replication (10^−10^)	Calcium ion binding (10^−6^)	RAD51AP1 PRIM1 (<10^−5^) KNTC1 NUP107 BARD1 RRM2 CDK1 HAT1 DEK HELLS (<0.001)	GIGYF2 SORBS2 TANC1 (0.02) SLITRK6 STAT2 (0.03) PTPRS FUNDC2 TMX2 LOXL3 TM9SF2 (0.05)
	Cell division (10^−9^)	Carbohydrate binding (10^−5^)		
	Mitotic nuclear division (10^−9^)	Endoplasmic reticulum lumen (10^−5^)		
	Sister chromatid cohesion (10^−9^)	Extracellular region (10^−5^)		
	Mitotic sister chromatid segregation (10^−9^)	Golgi membrane (10^−5^)		

^1^ False discovery rate (fdr) using likelihood ratio test.

## Supplementary Materials

The following are available online at http://www.mdpi.com/2079-7737/7/2/23/s1. Supplementary Figure S1: Relative activity of cellular programs of CREB, LDH and glycolysis along PT.
