# Supplementary material for: Pseudotime Dynamics in Melanoma Single-Cell Transcriptomes Reveals Different Mechanisms of Tumor Progression
Source: Biology (Basel). 2018 Apr 3;7(2):23. doi: 10.3390/biology7020023 (PMC6022966; doi:10.3390/biology7020023)
Supplement: Supplementary file 1 [file biology-07-00023-s001.pdf]

biology

MDPI

Article

Pseudotime Dynamics in Melanoma Single-Cell Transcriptomes Reveals Different Mechanisms of Tumor Progression

Henry Loeffler-Wirth [1], Hans Binder [1], Edith Willscher [1], Tobias Gerber [2] and Manfred Kunz [3,*]

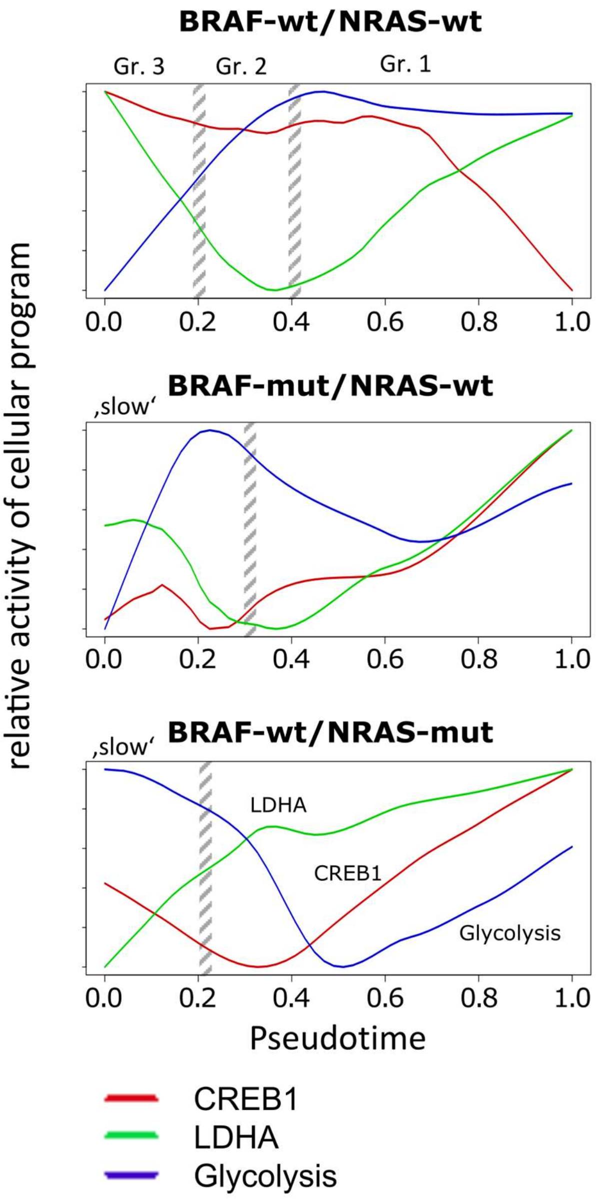

Supplementary Figure 1. Relative activity of cellular programs of CREB, LDH and glycolysis along PT. Melanoma short-term cultures were analyzed by single-cell RNA-seq and subjected to PT analysis using the Wanderlust algorithm and analysis of relative reactivity profiles of CREB1, LDH and glycolysis. Vertical streaked bars indicate the boundaries of the three different cell subpopulations in wt/wt cells or the two different cell populations in BRAF-mut/NRAS-wt and BRAF-wt/NRAS-mut cells as indicated in Figure 1C.
